# Stimulation of Dectin-1 and Dectin-2 during Parenteral Immunization, but Not Mincle, Induces Secretory IgA in Intestinal Mucosa

**DOI:** 10.1155/2018/3835720

**Published:** 2018-03-14

**Authors:** Alina S. Dzharullaeva, Amir I. Tukhvatulin, Alina S. Erokhova, Alina S. Bandelyuk, Nikita B. Polyakov, Andrey I. Solovyev, Natalia A. Nikitenko, Dmitry V. Shcheblyakov, Boris S. Naroditsky, Denis Y. Logunov, Alexander L. Gintsburg

**Affiliations:** N. F. Gamaleya National Research Center for Epidemiology and Microbiology, Gamaleya str.18, Moscow 123098, Russia

## Abstract

Induction of a robust and long-lived mucosal immune response during vaccination is critical to achieve protection against numerous pathogens. However, traditional injected vaccines are generally poor inducers of mucosal immunity. One of the effective strategies to improve vaccine efficacy is incorporation of adjuvant molecules that enhance and polarize adaptive immune reactions. Effects of Syk-coupled lectin receptor agonists as adjuvants to induce mucosal immune reactions during parenteral immunization are not fully studied. We now report that the agonists trehalose-6,6-dibehenate (TDB), curdlan, and furfurman, which stimulate Dectin-1, Dectin-2, and Mincle, respectively, activate transcription factors (NF-*κ*B, NFAT, and AP-1) to various extents in murine RAW 264.7 macrophages, even though similar pathways are activated. The agonists also elicit differential expression of maturation markers in bone marrow-derived dendritic cells, as well as differential cytokine secretion from these cells and from splenic mononuclear cells. *In vivo* assays also show that agonists of Dectin-1 and Dectin-2, but not Mincle, induce heavy IgA secretion in intestinal mucosa even when delivered parenterally. Strikingly, this effect appears to be formulation-independent. Collectively, the data suggest that adjuvants based on Dectin-1 and Dectin-2 agonists may significantly improve the efficacy of parenteral vaccines by inducing robust local immune reactions in intestinal mucosa.

## 1. Introduction

Vaccination has greatly reduced the burden of infectious diseases, preventing around 2-3 million annual deaths worldwide [1]. However, existing vaccines prevent only approximately 10% of all life-threatening infections. Thus, new vaccines, as well as novel approaches to effectively induce protective immunity, are urgently needed.

Most pathogens use epithelial barriers in the respiratory (*Mycobacterium tuberculosis* [2], adenovirus [3], and coronavirus [4]), gastrointestinal (*Salmonella* [5], *Helicobacter pylori* [6], and *Escherichia coli* [7]), and urogenital tract (*Chlamydia* [8], human papillomavirus [9], and human immunodeficiency virus [10]) as ports of entry. Accordingly, elements of mucosal immunity, including antigen-specific secretory IgA, T cells, cytokines, and antimicrobial peptides, promote protection against these infections [2, 11]. However, traditional injected vaccines are generally poor inducers of mucosal immunity and are therefore less effective against mucosal infections than mucosal vaccines [12]. Hence, it is important to investigate the induction and maintenance of mucosal immunity in order to generate effective vaccines against multiple pathogens.

It is known that pattern recognition receptors (PRRs) play essential role in the formation of immune defense on mucosal surfaces [13]. While the beneficial effect of PRR signaling for mucosal protective immunity has been established, the ability of PRR agonist-based adjuvants to promote local mucosal immune reactions during parenteral vaccination has not been extensively investigated. Thus, subcutaneous priming followed by airway booster immunization with CAF01, a clinically approved adjuvant, loaded with the C-type lectin (CLR) agonist trehalose-6,6-dibehenate (TDB), was recently demonstrated to induce strong immune reactions in the airway mucosa, including enhanced formation of Th17 cells and abundant IgA secretion [14, 15]. However, mucosal adjuvants based on other CLR agonists remain largely uncharacterized.

Hence, we investigated the ability of TDB, curdlan, and furfurman, which stimulate Mincle, Dectin-1, and Dectin-2, respectively, to elicit both mucosal and systemic immune reactions via several transcription factors (NF-*κ*B, NFAT, and AP-1) and cellular pathways (cytokine secretion and maturation of dendritic cells). Head-to-head comparison using reporter cell lines showed that the agonists activate transcription factors to varying extents. *In vitro* studies showed that TDB and curdlan elicit production of mixed Th1/Th17 cytokines (IFN*γ*, TNF*α*, IL-12, IL-6, and IL-23), whereas furfurman predominantly induces production of Th17 cytokines (IL-6, IL-17A, and IL-23). Finally, we demonstrate for the first time that subcutaneous prime-boost immunization with curdlan and furfurman, but not with TDB, boosts IgA production in intestinal mucosa after parenteral immunization. Collectively, these results highlight Dectin-1 and Dectin-2 agonists as potential adjuvants to induce local mucosal immunity during parenteral immunization.

## 2. Materials and Methods

### 2.1. Lentiviral Stock Preparation

293 T cells were used for the preparation of lentiviral stocks. Cells were transfected with vectors using Lipofectamine-Plus Reagent (Invitrogen, USA). Two helper plasmids, pCMV*Δ*R8.2 and pVSV-G (Addgene, USA), were cotransfected along with the experimental plasmids: pGF-NF-*κ*B-mCMV-EF1-puro, pGF-AP-1-mCMV-EF1-puro, and pGF-NFAT-mCMV-EF1-puro, correspondingly. Lentiviral plasmids carrying a transcriptional factor- (NF-*κ*B-, AP-1-, and NFAT-) inducible luciferase reporter genes pGF-NF-*κ*B-mCMV-EF1-puro, pGF-AP-1-mCMV-EF1-puro, and pGF-NFAT-mCMV-EF1-puro were obtained from System Biosciences (USA). Supernatants containing infectious viral particles were harvested 24 and 48 h posttransfection, pooled, and filtered.

### 2.2. Construction of NF-*κ*B-, AP-1-, and NFAT-Reporter Cell Lines

RAW-Blue cells derived from RAW 264.7 murine macrophages that were obtained from InvivoGen were used as a parental cell line. Cell culture medium containing lentiviruses with three different reporters LV-NF-*κ*B-Luc, LV-NFAT-Luc, and LV-AP-1-Luc was used to the transduction of target cells to obtain three separate reporter lines: RAW-NF-*κ*B-Luc, RAW-NFAT-Luc, and RAW-AP-1-Luc, correspondingly. For the next 5 days, puromycin (0.5 *μ*g/mL) was added to each cell culture medium for the selection of transduced cells.

### 2.3. Luciferase Reporter Assay

Reporter cells (RAW-NF-*κ*B-Luc, RAW-NFAT-Luc, and RAW-AP-1-Luc) were cultured in DMEM medium (GE Healthcare, USA) supplemented with 10% fetal bovine serum (FBS, Thermo Fisher Scientific, USA), 50 U/mL penicillin, 50 *μ*g/mL streptomycin, 2 mM glutamine, and 0.1 M NaHCO_3_ (all PanEco, Russia) at 37°C with 5% CO_2_. We used the protocol described in our previous work [16]; namely, experiment cells were seeded at 1 × 10^5^ cells/well in 96-well plates (100 *μ*L/well). The next day, C-type lectin receptor agonists were added to the wells to a final concentration of 20 *μ*g/mL, 4 *μ*g/mL, and 1 *μ*g/mL. Eight hours later, to detect luciferase activity, 100 *μ*L of Bright-Glo Luciferase Assay Buffer containing luciferin substrate (Promega, USA) was added to each well. Luminescence was measured in relative units using a Synergy H4 Hybrid Reader (BioTek, Germany).

### 2.4. Mice

Female 8–10 weeks old SPF C57BL/6 mice were purchased in Nursery “Pushchino” (Institute Bioorganic Chemistry, Russia). The mice were fed a completely pelleted laboratory chow and had access to food and water ad libitum. The mice were housed in cages with 5–10 mice/cage in ISOcage isolator system (Tecniplast, Italy). All of the experimental procedures conformed to the Guide for the Care and Use of Laboratory Animals published by the National Institutes of Health (NIH publication number 85–23, revised 1996) and approved by local ethic committee of N.F. Gamaleya National Research Center for Epidemiology and Microbiology. Mice were euthanized by CO_2_ overdose prior to autopsy.

### 2.5. Dendritic Cell Culture

Bone marrow-derived dendritic cells (BMDCs) from C57BL/6 mice were differentiated from proliferating mouse bone marrow progenitors through induction with 20 ng/mL granulocyte macrophage colony-stimulating factor (GM-CSF) (R&D Systems, USA) over 8 days as described [17]. Briefly, mice were euthanized by CO_2_ overdose. Femurs and tibias were collected in phosphate buffered saline (PBS) solution (Sigma-Aldrich, USA). The muscles were removed with a scalpel. Then, epiphyses were cut off with scissors. The bone marrow was flushed out with 2-3 mL of RPMI medium in a syringe with a 25G needle and resuspended. All bone marrow cells were collected and washed twice with PBS. The bone marrow cells were cultured in Petri dishes containing approximately 5 × 10^5^ cells/mL in 10 mL total volume. The cells were maintained at 37°C with 5% CO_2_ in complete RPMI medium with 10% heat inactivated fetal bovine serum (FBS, Thermo Fisher Scientific, USA), 0.1 M NaHCO_3_, 0.05 mM mercaptoethanol (Thermo Fisher Scientific, USA), essential and non-essential amino acids (PanEco, Russia), 20 ng/mL GM-CSF, 2 mM glutamine, 100 U/mL penicillin, and 100 *μ*g/mL streptomycin, 1 mM sodium pyruvate, and 10 mM HEPES pH 7.4 (all PanEco, Russia). After 24 hours, 10 mL of fresh media were added to Petri dishes. On days 3, 5, and 7, half of the medium in each dish was replaced with 10 mL of fresh medium. On days 7–10, the percentage of CD11c^+^ cells in the cultures was 70–80%.

### 2.6. Cytokine and Chemokine Analysis

BMDCs or splenocytes were seeded in 96-well plates at 1 × 10^5^ cells per well (100 *μ*L/well) in complete RPMI medium. Cells were treated with CLR agonists (final concentration of 10 *μ*g/mL) in triplicates. Twenty-four hours after treatment, plates were centrifuged at 400 ×g for 10 min, and the culture supernatants were collected. Levels of 26 cytokines and chemokines (IL-1*α*, IL-1*β*, IL-2, IL-3, IL-4, IL-5, IL-6, IL-9, IL-10, IL-12 p40, IL-12 p70, IL-13, IL-17A, eotaxin (CCL11), G-CSF, GM-CSF, IFN*γ*, KC (CXCL1), MCP-1 (CCL2), MIP-1*α* (CCL3), MIP-1*β* (CCL4), RANTES (CCL5), and TNF*α*, as well as TGF-*β*1, TGF-*β*2, and TGF-*β*3) were measured in the prepared supernatants using bead-based assay (23-plex and 3-plex TGF-*β* Group Bio-Plex Pro kits) according to the manufacturer's instructions (Bio-Rad, USA).

### 2.7. Flow Cytometric Analysis of BMDCs

BMDCs were seeded in 24-well plates at 1 × 10^6^ cells per well in 1 mL RPMI complete medium. TDB, curdlan, and furfurman were added to the cells at concentration of 10 *μ*g/mL. After 24 hours, the cells were collected, washed twice in PBS, and stained in Staining Buffer (BD Biosciences, USA) for 30 min at 4°C for surface markers with fluorescently labeled antibodies: anti-CD11c-PE-CF594 (clone HL3), anti-MHCII-PE (clone 2G9), anti-CD80-BV421 (clone 16-10A1), anti-CD83-APC (clone Michel-19), and anti-CD86-AF700 (clone GL1) (all BD Biosciences, USA). Cells were analyzed on a FACS Aria III instrument (BD Biosciences, USA).

### 2.8. Preparation of Vaccine Compositions and Immunization

To obtain vaccine compositions, ovalbumin (Sigma-Aldrich, USA) (10 *μ*g/dose) was mixed with individual CLR agonists: TDB, curdlan, and furfurman (all, InvivoGen, USA) (50 *μ*g/dose) in 100 *μ*L sterile PBS. Interactions between antigen and individual CLR agonists were measured by surface plasmon resonance imaging. Squalene-based vaccine formulations were made by thorough mixing ovalbumin antigen (10 *μ*g/dose) and CLR agonists (50 *μ*g/dose) with 10 *μ*L squalene (Sigma-Aldrich, USA) and 15 *μ*L of 10% Tween 80 (PanReac AppliChem, Germany). The volume of each formulation was adjusted with PBS to a total of 200 *μ*L/dose. Mixtures were further sonicated (A—20%, 30 sec) (Branson, USA). The emulsions were stable for two weeks (the particle size was about 350 nm and did not change). Stability was controlled in the Zetasizer Nano ZS (Malvern, UK). Mice were immunized by the subcutaneous route (s.c., volume of 100 *μ*L) at the base of the tail. Two immunizations were given with 2 weeks interval.

### 2.9. Preparation of Mouse Splenic Mononuclear Cells

Spleens from intact mice were harvested and dissociated using scissors and 100 *μ*m nylon cell strainer (Falcon, USA). Splenocytes were purified by Ficoll 1.09 g/mL (PanEco, Russia) density gradient centrifugation (400 ×g, 30 min) and washed twice in PBS. Mononuclear cells were counted and normalized using an automated cell counter TC20 (Bio-Rad, USA).

### 2.10. Analysis of T Cell Responses

Mice were euthanized 14 days after the second immunization, and splenic mononuclear cells were isolated according to the above-mentioned method. For analysis of antigen-specific T cell proliferation response, cells were stained with carboxyfluorescein succinimidyl ester (CFSE) tracer kit (Invitrogen, USA) as described previously [18] and seeded in 96-well plates at 2 × 10^5^ cells per well. Cells were restimulated with whole ovalbumin antigen at 1 *μ*g/mL and cultured in complete RPMI medium at 37°C in 5% CO_2_. 72 hours later, cells were harvested, washed in PBS, and then stained using BD Pharmingen Mouse T-Lymphocyte Subset Antibody Cocktail (anti-CD3, anti-CD4, and anti-CD8) with corresponding isotype controls for 20 min at 4°C, in Staining Buffer (all, BD Biosciences, USA) according to the manufacturer's instructions. Cells were analyzed on a FACS Aria III instrument (BD Biosciences, USA).

### 2.11. Blood Serum and Lavage Samples Collection

Two weeks after the second immunization, mice were euthanized by CO_2_ overdose. Blood samples were taken using a syringe from the heart, after thoracotomy. After collection, blood samples were incubated at 37°C for 30 min and centrifuged (300 ×g, 10 min). Supernatant (serum) was immediately aliquoted and stored at −80°C. Bronchoalveolar lavage (BAL) specimens were obtained using 23 gauge lavage tubes by infusing 0.5 mL of PBS into the lungs via the trachea, followed by aspiration of this fluid into a syringe. For collection of intestinal lavage (IL) samples, small intestine was removed and carefully filled with 3 mL of PBS containing Sigmafast Protease Inhibitor Cocktail (Sigma, USA) using a syringe. After 10 min incubation, solution was transferred into the 1.5 mL conical tubes (Eppendorf, Germany). Tubes were placed in ice and sonicated for 20 minutes in ultrasonic water bath (Elma Schmidbauer, Germany). Then, samples were centrifuged for 10 minutes at 1800 rpm at 4°C. After obtaining BAL and IL, samples were immediately aliquoted and stored at −80°C until analysis.

### 2.12. Measurement of Antigen-Specific Antibody Titers in Serum and Lavage Samples from Immunized Mice

Total ovalbumin-specific IgG and IgG isotype titers in blood serum and IgA titers in intestinal and alveolar lavage fluid were measured in samples collected from the mice 14 days after the second immunization. 96-well microtiter plates (Costar, USA) were coated with 100 *μ*L per well of 10 *μ*g/mL ovalbumin solution in carbonate buffer pH 9.6 overnight at 4°C. On the next day, plates were washed three times with PBS containing 0.05% Tween 20 (TPBS) and blocked with 5% (w/v) non-fat milk in TPBS for 30 minutes at 37°C and 300 rpm. Individual mouse sera or lavage fluid from five mice per group was analyzed individually in serial twofold dilutions prepared in TPBS containing 5% (w/v) non-fat milk. Samples (100 *μ*L per well) were then added to the coated, washed, and blocked ELISA plates. After incubating the plates with samples for 1 hour at 37°C, the plates were washed four times with TPBS, and 100 *μ*L horseradish peroxidase-labeled secondary antibodies were added to each well. Secondary HRP-conjugated antibodies were goat polyclonal antibodies to mouse total IgG (GE Healthcare, Germany), goat polyclonal antibodies to mouse IgG1, IgG2, IgG2b, and IgG3, and goat polyclonal antibodies to mouse IgA (all, Abcam, UK) and were diluted 1 : 5000 in TPBS containing 5% (w/v) non-fat milk. The plates were incubated for 1 hour at 37°C and then washed five times with TPBS. For detection of bound antibodies, 100 *μ*L of the peroxidase substrate 3,3′,5,5′-tetramethylbenzidine (TMB) was added to each well. The plates were incubated at RT for 10–40 min for color development. The reaction was stopped using 4 M H_2_SO_4_. The optical density (OD) in each well was measured at 450 nm using a spectrophotometric plate reader Multiscan FC (Thermo Fisher Scientific, USA). We followed the methods of [19] and the measurement of antigen-specific antibody titers in serum in Materials and Methods.

### 2.13. Mass Spectrometer Analysis

Molecular weight (Mw) of TDB, curdlan, and furfurman was determined using MALDI-TOF mass spectrometer ultrafleXtreme (Bruker Daltonics, Germany). Positive ions were detected in а reflectron mode using the following voltages: IS1 20.12 kV and IS2 17.82 kV; on lenses, 7.47 kV; reflectron, Ref1 21.07 kV and Ref2 10.80 kV; ions were detected in the range m/z from 120 to 5000 Th and in the linear mode in the range from 2000 to 20,000 Th at the following voltages: on the ion source IS1 20.12 kV and IS2 19.12 kV; on the lenses 4.48 kV.

### 2.14. Surface Plasmon Resonance Imaging

The interaction of CLR agonists with ovalbumin was determined by surface plasmon resonance (SPR) using a Biacore 3000 (GE Healthcare, Uppsala, Sweden) equipped with a research-grade CM5 sensor chip (BR100012, GE Healthcare) at a temperature of 25°C. Ovalbumin was immobilized at amount of 30,000 response units (RUs) in 10 mM acetate buffer pH = 5.0 on a CM5 sensor chip using the amine coupling kit supplied by the manufacturer (GE Healthcare, Sweden). TDB, curdlan, and furfurman were used at concentration of 100 *μ*g/mL, 50 *μ*g/mL, and 25 *μ*g/mL. As a control of nonspecific binding of agonist with antigen, we used CpG oligonucleotide ODN1826 (InvivoGen, USA) at a concentration of 100 *μ*g/mL. PRR agonists were dissolved in sterile deionized water containing 10% v/v DMSO. Analyses were performed in 10 mM HBS-EP running buffer (HEPES buffered saline containing 150 mM NaCl, 3 mM EDTA, and 0.005% surfactant P20, pH 7.4) at a flow rate of 30 *μ*L/min. Calculations were performed using BIAevaluation software (GE Healthcare, Uppsala, Sweden) with reference-subtracted fitting. Representative images of SPR are shown in Figure S2.

### 2.15. Statistical Analysis

All experiments were performed three times unless otherwise specified, and data are expressed as the mean ± SD of the values from all experiments. Statistical significance was assessed using a two-tailed unpaired Mann–Whitney *t*-test with a threshold set at *p* < 0.05.

## 3. Results

### 3.1. Stimulation of Mincle, Dectin-1, and Dectin-2 Elicits Distinct Transcriptional Responses in Murine Macrophages

**Figure 1:**
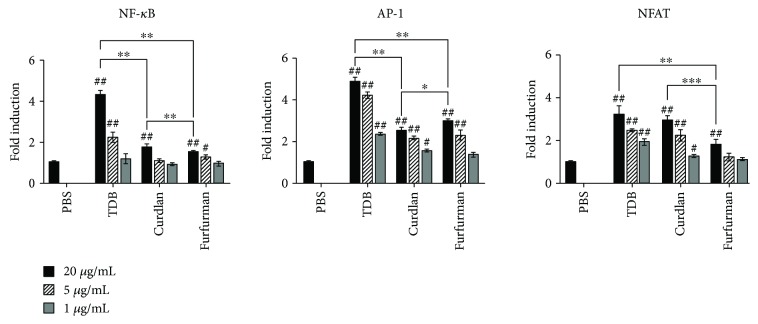
NF-*κ*B-, AP-1-, and NFAT-dependent luciferase reporter gene expressions in RAW-NF-*κ*B-Luc, RAW-NFAT-Luc, and RAW-AP-1-Luc macrophages, correspondingly, treated for 8 h with the indicated doses (*μ*g/mL) of Mincle, Dectin-1, and Dectin-2 agonists. Data are mean ± SD fold increase over PBS-treated cells from three independent experiments, each performed in duplicates. Data were compared using the two-tailed unpaired Mann–Whitney *t*-test. # indicates significant differences in luciferase expression between control (PBS-treated) and TDB-, curdlan-, or furfurman-treated cells (^#^ *p* < 0.05; ^#^^#^ *p* < 0.005). ∗ indicates significant differences in luciferase expression between Mincle-, Dectin-1-, and Dectin-2-stimulated cells (^∗^ *p* < 0.05; ^∗∗^ *p* < 0.005; ^∗∗∗^ *p* < 0.0005).

Stimulation of Syk-coupled CLRs was previously shown to activate several proinflammatory transcription factors, including NF-*κ*B, NFAT, and AP-1, in response to microbial infection and tissue damage [20–22]. However, the transcriptional responses triggered by different CLRs have not been directly compared. Thus, we used curdlan, furfurman, and TDB to investigate AP-1-, NFAT-, and NF-*κ*B-dependent expressions of an exogenous luciferase reporter gene in three sublines derived from RAW 264.7 macrophages, expressing endogenous Dectin-1, Dectin-2, and Mincle. Maximum NF-*κ*B- and AP-1-dependent luciferase expressions were observed in cells exposed to 20 *μ*g/mL TDB, with expression increasing 4.1- and 5.0-fold relative to untreated cells, respectively (Figure 1). The same doses of curdlan and furfurman resulted in 1.8- and 1.6-fold increase in NF-*κ*B-dependent expression and in 2.5- and 3.0-fold increase in AP-1-dependent expression. On the other hand, maximum NFAT activation was achieved with TDB (3.0-fold) and curdlan (2.9-fold). In contrast, furfurman activated NFAT (1.8-fold) to a significantly lower degree. Normalization of expression from individual promoters to total expression also highlights the differential transcriptional response to each agonist (Figure S1). Collectively, the data imply that stimulation of the CLRs Mincle, Dectin-1, and Dectin-2 results in different transcriptional profiles, despite engaging the same signaling cascades.

### 3.2. Stimulation of Mincle, Dectin-1, and Dectin-2 Differentially Activates Bone Marrow-Derived Dendritic Cells

**Figure 2:**
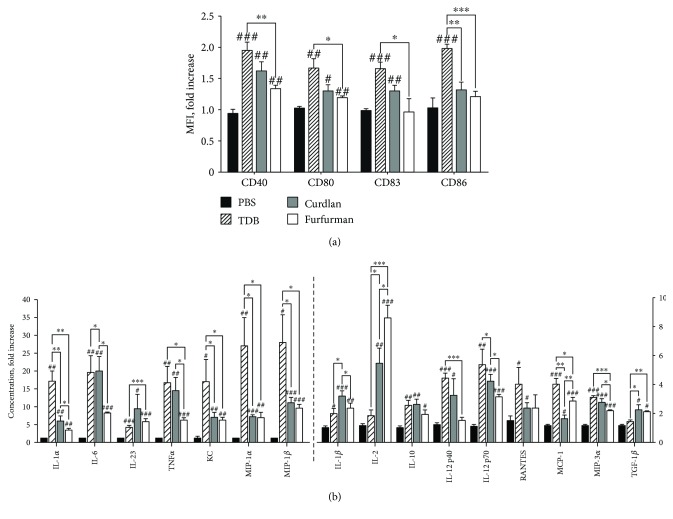
Response of bone marrow-derived dendritic cells to Mincle, Dectin-1, and Dectin-2 agonists. Cells were harvested on day 8 of culture, seeded in 24-well plates (2 × 10^5^ cells/well), and incubated for 24 h with 10 *μ*g/mL of Mincle, Dectin-1, and Dectin-2 agonists. (a) Expression of the maturation markers CD40, CD80, and CD86 was assessed by flow cytometry of 10^6^ CD11c^+^ MHCII^+^ cells. Data are mean fluorescence fold increase over PBS-treated cells from two independent experiments with triplicates. Means were compared using the two-tailed unpaired Mann–Whitney *t*-test. Error bars indicate standard deviations. ^#^ *p* < 0.05; ^#^^#^ *p* < 0.01; ^#^^#^^#^ *p* < 0.005 versus PBS-treated cells. ^∗^ *p* < 0.05; ^∗∗^ *p* < 0.01; ^∗∗∗^ *p* < 0.005 between Mincle-, Dectin-1-, and Dectin-2-stimulated cells. (b) Cytokines were quantified by bead-based immunoassay in media collected 24 h after addition of agonists. Data are mean fold change relative to PBS-treated cells in three independent experiments with duplicates. Data were compared using the two-tailed unpaired Mann–Whitney *t*-test. Error bars indicate standard deviations. ^#^ *p* < 0.05; ^#^^#^ *p* < 0.01; ^#^^#^^#^ *p* < 0.005 versus PBS-treated cells. ^∗^ *p* < 0.05; ^∗∗^ *p* < 0.01; ^∗∗∗^ *p* < 0.005 between Mincle-, Dectin-1-, and Dectin-2-stimulated cells.

Based on the observed differences in activity levels of NF-*κ*B, AP-1, and NFAT transcription factors, we hypothesized that CLR agonists may also affect downstream immune reactions in different ways. As dendritic cells, which determine the magnitude and polarization of adaptive immunity [23], abundantly express CLRs, we quantified maturation markers in bone marrow-derived dendritic cells [24, 25] exposed for 24 h to 10 *μ*g/mL TDB, curdlan, and furfurman. Stimulation of Mincle with TDB increased expression of CD40, CD80, CD83, and CD86 by 1.95-, 1.7-, 1.65-, and 2.0-fold, respectively. Curdlan also enhanced expression of CD40 (1.6-fold), CD80 (1.3-fold), and CD83 (1.3-fold), whereas furfurman induced expression of CD40 (1.3-fold) and CD80 (1.2-fold) only (Figure 2(a)). In addition, the agonists also induced to various extents the secretion of 16 cytokines and chemokines, out of the 26 we tested (Figure 2(b)). In particular, TDB elicited the strongest expression of IL-1*α* (17.1-fold), KC (16.4-fold), MIP-1*α* (27.0-fold), MIP-1*β* (29.9-fold), TNF*α* (16.7-fold), IL-12 p40 (4.4-fold), IL-12 p70 (5.3-fold), RANTES (4.0-fold), MCP-1 (3.9-fold), and MIP-3*α* (3.0-fold). On the other hand, IL-1*β* (3.2-fold), IL-6 (20.0-fold), and IL-23 (9.5-fold increase) were most strongly induced in the presence of curdlan, while IL-2 (8.7-fold) was most effectively stimulated by furfurman. Only curdlan and furfurman stimulated TGF-*β*1 expression (2.0-fold) but to a comparable extent.

### 3.3. Stimulation of Mincle, Dectin-1, and Dectin-2 Induces Distinct Cytokine Profiles in Mouse Splenic Mononuclear Cells

**Figure 3:**
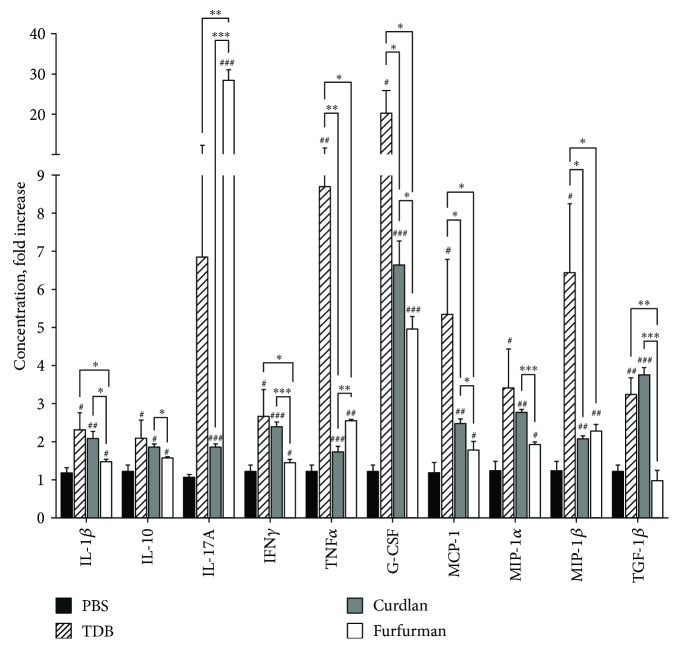
Cytokine and chemokine expression in murine splenic mononuclear cells after Mincle, Dectin-1, and Dectin-2 stimulation. Splenic mononuclear cells were isolated from C57/Bl6 mice, seeded in 96-well plates at 2 × 10^5^ cells/well, and stimulated for 48 h with 10 *μ*g/mL Mincle, Dectin-1, and Dectin-2 agonists. Cytokines were quantified in media by bead-based immunoassay. Data are mean fold change relative to PBS-treated cells in three independent experiments with duplicates, and error bars indicate standard deviations. Data were compared using the two-tailed unpaired Mann–Whitney *t*-test. ^#^ *p* < 0.05; ^#^^#^ *p* < 0.01; ^#^^#^^#^ *p* < 0.005 versus PBS-treated cells. ^∗^ *p* < 0.05; ^∗∗^ *p* < 0.01; ^∗∗∗^ *p* < 0.005 between Mincle-, Dectin-1-, and Dectin-2-stimulated cells.

Polarization of adaptive immunity is also driven by cytokines and chemokines secreted by lymphocytes, among which different Th subsets have been identified based on signature cytokine profiles [26]. To evaluate cellular immune reactions similar to those *in vivo* after injection, we quantified 26 cytokines and chemokines 48 h after stimulating splenic naïve mononuclear cells with 10 *μ*g/mL TDB, curdlan, and furfurman. Strikingly, secretion of 10 cytokines and chemokines was enhanced relative to control cells (Figure 3), although the effects on secretion depended on the agonist. For example, TNF*α* was maximally induced (8.7-fold) with TBD, along with G-CSF (19.4-fold), MCP-1 (5.3-fold), and MIP-1*β* (6.4-fold). Similarly, curdlan induced IFN*γ* (2.4-fold) more effectively than the other agonists. TDB and curdlan also induced IL-1*β* (2.3-fold and 2.0-fold) and TGF-*β*1 (3.2-fold and 3.7-fold) more strongly than furfurman. Surprisingly, furfurman induced IL-17A (28.1-fold) more robustly than TDB (6.8-fold) and curdlan (1.8-fold) but did not affect any other cytokine or chemokine. Taken together, the data demonstrate that CLR agonists elicit a strong proinflammatory response but to various extents.

### 3.4. Mincle, Dectin-1, and Dectin-2 Agonists Induce Strong Cellular Adaptive Immune Response *In Vivo*

As TDB, curdlan, and furfurman mature bone marrow-derived dendritic cells, as well as induce cytokine secretion from such cells and from mononuclear cells, these agonists may induce strong adaptive immune response *in vivo* when used as adjuvants. Therefore, we evaluated the immune response to 10 *μ*g ovalbumin prepared with or without 50 *μ*g agonists. We note that the immune response to these complexes was evaluated in the absence of additional carriers, for example, alum, squalene, and others, since these carriers may greatly influence the immune response, such as by shifting Th1/2/17 polarization [27].

We detected strong interaction between ovalbumin and CLR agonist molecules (*K*_D_ values were 2.35 × 10^−10^ M, 1.4 × 10^−9^ M, and 6.3 × 10^−8^ M for OVA-TDB, OVA-Curdlan, and OVA-Furfurman, resp.), as measured by surface plasmon resonance (Figure S2). ODN1826, a CpG oligonucleotide, was used as negative control to measure nonspecific binding. The mean diameter and surface charge (Z-potential) of ovalbumin-agonist complexes were also evaluated (Table S1). *K*_D_ values and the observed increase in mean diameters of antigen and CLR agonist mixtures comparing to individual components suggest that stable complexes were formed.

**Figure 4:**
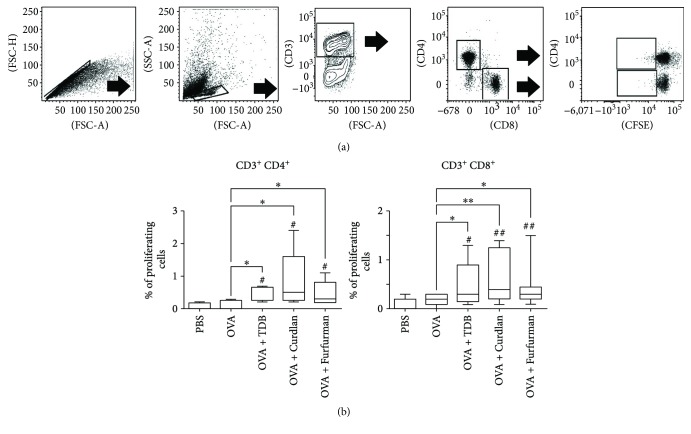
Mincle, Dectin-1, and Dectin-2 agonists induce comparable antigen-specific CD4^+^ and CD8^+^ T cell response. Mice were immunized twice by subcutaneous injection of ovalbumin alone or in combination with TDB, curdlan, or furfurman. Naïve mice were used as negative control. Splenocytes were harvested 14 days after the second immunization, labeled with CFSE, restimulated with 1 *μ*g/mL ovalbumin for 72 h, stained with fluorescently tagged antibodies against CD3, CD4, and CD8, and analyzed by flow cytometry. (a) Gating strategy to quantify proliferating CD4^+^ and CD8^+^ T cells. (b) Minimum, maximum, and median of the abundance of ovalbumin-specific proliferating CD4^+^ and CD8^+^ T cells from two independent experiments with 5 mice per group per experiment. Data were compared using the two-tailed unpaired Mann–Whitney *t*-test. ^#^ *p* < 0.05; ^#^^#^ *p* < 0.01 relative to control group. ^∗^ *p* < 0.05; ^∗∗^ *p* < 0.01 between Mincle-, Dectin-1-, and Dectin-2-stimulated cells.

The mixtures were administered to mice in two subcutaneous injections two weeks apart. Splenic mononuclear cells were then collected two weeks after the second immunization and analyzed by CFSE fluorescence proliferation assay 72 hours after antigen restimulation *in vitro*. The gating strategy to quantify proliferating, antigen-specific CD4^+^ and CD8^+^ T cells is illustrated in Figure 4(a), and the minimum, maximum, and median abundance of these cells is plotted in Figure 4(b). The mean proliferative response was higher in mice immunized with soluble ovalbumin only (0.16% CD4^+^ and 0.18% CD8^+^) but not to a significant extent in comparison to naïve mice (0.08% CD4^+^ and 0.1% CD8^+^). On the other hand, adjuvantation with CLR agonists resulted in increased proliferation of both CD4^+^ and CD8^+^ T cells in response to restimulation. In comparison to cells from mice immunized with ovalbumin only, cells from mice immunized with ovalbumin and TDB contained an additional 0.5% each of CD4^+^ and CD8^+^ T cells. Similarly, cells from mice immunized with ovalbumin and curdlan contained 0.84% more CD4^+^ cells and 0.7% more CD8^+^ T cells, while cells from mice immunized with ovalbumin and furfurman produced 0.5% additional CD4^+^ cells and 0.4% additional CD8^+^ T cells. Comparison of T cell proliferative response between compositions containing CLR agonists did not show significant differences.

Observed data highlight the potential of Dectin-1, Dectin-2, and Mincle agonists as adjuvants to enhance the T cell response.

### 3.5. Mincle, Dectin-1, and Dectin-2 Agonists Induce Differential IgG Profiles *In Vivo*

**Figure 5:**
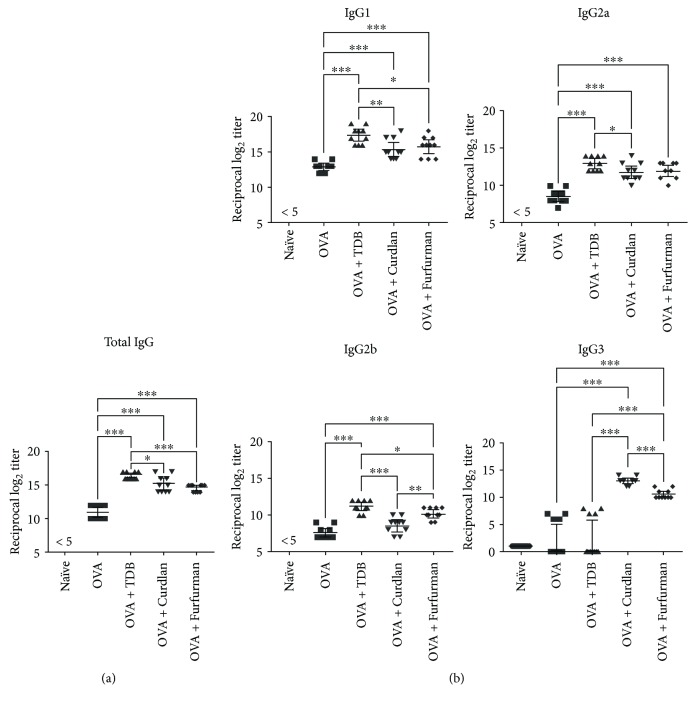
Mincle, Dectin-1, and Dectin-2 agonists induce antigen-specific IgG in mice. Mice were immunized twice by subcutaneous of ovalbumin alone or in combination with TDB, curdlan, or furfurman. Naïve mice were used as negative control. (a) Serum total IgG and (b) IgG1, IgG2a, IgG2b, and IgG3 were determined by ELISA 14 days after the second immunization. Data are individual log_2_ antibody titers from two independent experiments (5 mice per group per experiment), with geometric mean and 95% confidence interval indicated. ^∗^ *p* < 0.05; ^∗∗^ *p* < 0.005; ^∗∗∗^ *p* < 0.0005 relative to control group by Mann–Whitney *t*-test.

To evaluate the humoral adaptive immune response induced by compositions containing antigen and individual CLR agonist, blood was collected 14 days after the last injection from mice immunized as above and assayed for total IgG and IgG isotypes. Naïve mice were used as control. Total ovalbumin-specific IgG was significantly more abundant in mice injected with ovalbumin-CLR agonist mixtures than in mice immunized with soluble ovalbumin only (Figure 5(a)). The humoral response was strongest and significantly higher in mice immunized with ovalbumin and TDB (geometric mean reciprocal IgG titer: 84,500), in comparison to mice immunized with ovalbumin and curdlan (36,760) or with ovalbumin and furfurman (24,250). Moreover, adjuvantation with TDB induced the highest levels of ovalbumin-specific IgG1 (geometric mean reciprocal IgG1 titer: 188,162) and IgG2b (2300), although IgG3 was not significantly elevated (<125; Figure 5(b)). IgG2a titers were comparable between mice immunized with ovalbumin and TDB (8000) or furfurman (4000), but IgG2b titers were higher in the latter (1150) than in the former (380). Conversely, IgG3 antibodies were more abundant in the OVA-Curdlan group (8000) than in the OVA-Furfurman group (1515). Collectively, the results indicate that adjuvantation with TDB, curdlan, and furfurman induces strong humoral responses, although the resulting IgG profiles vary.

### 3.6. Dectin-1 and Dectin-2 Agonists, but Not Mincle Agonist, Induce IgA in Intestinal Mucosa

**Figure 6:**
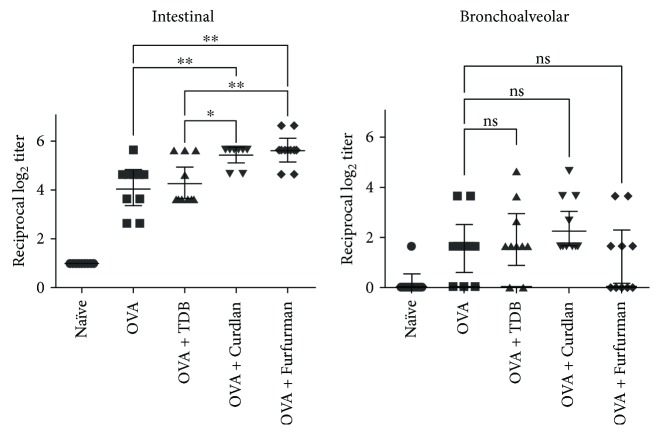
Dectin-1 and Dectin-2 agonists induce production of antigen-specific IgA in the small intestine. Mice were immunized twice by subcutaneous injection of ovalbumin alone or in combination with TDB, curdlan, or furfurman. Naïve mice were used as negative control. IgA titers were quantified by ELISA in (a) intestinal and (b) bronchoalveolar lavages 14 days after the second immunization. Data are individual log_2_ antibody titers from two independent experiments (5 mice per group per experiment), with geometric mean and 95% confidence interval indicated. ^∗^ *p* < 0.05; ^∗∗^ *p* < 0.005 relative to control group by Mann–Whitney *t*-test.

Since a strong mucosal immune response at the initial site of infection is an essential defense against pathogens, we investigated whether Dectin-1, Dectin-2, and Mincle agonists induce effective mucosal immunity. To this end, bronchoalveolar and intestinal lavage fluids were collected 14 days after the last immunization from mice immunized as described and assayed for antigen-specific IgA by ELISA (Figure 6). Adjuvantation with curdlan and furfurman significantly induced intestinal IgA (geometric mean IgA titer: 50 and 43.5), in comparison to ovalbumin alone (geometric mean IgA titer: 17.6). In contrast, adjuvantation with TDB increased intestinal IgA only slightly (geometric mean IgA titer: 28.7). It is worth to note that adjuvantation with lower doses of curdlan or furfurman (down to 10 *μ*g/dose) elicited significant production of IgA as well (data not shown), but adjuvantation with higher concentrations of TDB (up to 100 *μ*g/dose) did not (data not shown). Interestingly, parenteral administration of ovalbumin adjuvanted with CLR agonists did not significantly induce bronchoalveolar IgA. Taken together, the data suggest that adjuvantation with Dectin-1 and Dectin-2 agonists, but not with Mincle agonist, robustly induces IgA production in intestinal mucosa following parenteral immunization.

### 3.7. Dectin-1 and Dectin-2 Agonists Induce Intestinal IgA Independently of Formulation

It is known that number of vaccine formulation characteristics (such as chemical nature of the carrier, the size, shape, and surface charge of particles) may greatly affect immunogenicity of vaccine. For example, the changes in carrier nature may alter the biodistribution of the antigen, as well as its acquisition and processing by antigen-presenting cells, and thus may result in changes in the adaptive immune response [27]. To test whether the intestinal IgA response to Dectin-1 and Dectin-2 agonists depends on formulation, we tested squalene emulsions containing ovalbumin alone or with TDB, curdlan, and furfurman. Prepared formulations had similar physico-chemical characteristics, as assessed by average particle size and Z-potential (Table S2). Obtained data negate formulation discrepancies that could effect on immunogenicity except CLR agonist content. Serum IgG and intestinal IgA were measured two weeks after the second injection in mice immunized by the same prime-boost immunization as described.

**Figure 7:**
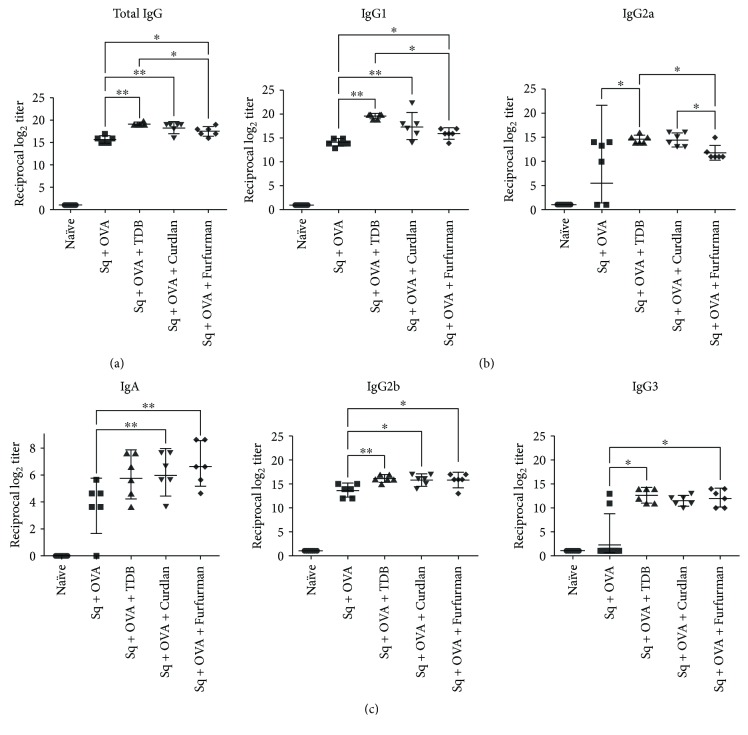
Serum IgG and intestinal IgA after prime-boost immunization using squalene-based vaccines containing ovalbumin alone or in combination with Mincle, Dectin-1, and Dectin-2 agonists. Mice were immunized twice by subcutaneous injection. Naïve mice were used as negative control. (a) Serum total IgG, (b) serum IgG1, IgG2a, IgG2b, and IgG3, and (c) intestinal IgA were quantified by ELISA 14 days after the second immunization. Data are individual log_2_ antibody titers from one representative experiment (6 mice per group), with geometric mean and 95% confidence interval indicated. ^∗^ *p* < 0.05; ^∗∗^ *p* < 0.005 relative to control group by Mann–Whitney *t*-test.

All squalene-based formulations induced IgG responses that were 2–4 log_2_ higher than antigen mixtures without carrier. Squalene-based formulations with TDB, curdlan, and furfurman resulted in significantly higher total IgG (574,000, 322,539, and 181,019, resp.) than ovalbumin alone (50,796; Figure 7(a)). Moreover, IgG1 and IgG2a were maximal in mice immunized with ovalbumin and TDB (182,749 and 25,398), although IgG2b and IgG3 were comparable among groups (Figure 7(b)). Evaluation of IgA titers in intestinal lavages showed that mice vaccinated with ovalbumin and curdlan (geometric mean IgA titer: 70.7) or furfurman (geometric mean IgA titer: 100) presented significantly increased IgA relative to mice vaccinated with ovalbumin only (geometric mean IgA titer: 15.8; Figure 7(c)). However, adjuvantation with TDB (geometric mean IgA titer: 4.6) did not induce mucosal IgA. Thus, stimulation of Dectin-1 and Dectin-2 during subcutaneous vaccination appears to elicit a mucosal IgA response independently of carrier present in vaccine formulation.

## 4. Discussion

Collectively, the gastrointestinal, urogenital, and respiratory tracts present, on average, 400 m^2^ of mucosal surface areas in the human body serving the major entry ports for numerous viral, bacterial, and fungal pathogens [28]. Thus, a vaccine should ideally enable to induce mucosal immunity to provide host defense at the point of entry of the infectious agent or at least limit such invasion until protective central immunity develops. Hence, elucidating the molecular and cellular mechanisms that drive local mucosal immunity is essential to develop more effective parenteral vaccines.

Recent developments suggest that effective vaccines require not only a suitable antigen and an appropriate delivery system that promotes lymphatic trafficking and antigen uptake by local antigen-presenting cells, but also immunostimulatory molecules that enhance the specific immune response and drive its polarization [29]. Accordingly, the discovery of pattern recognition receptors that strongly regulate both innate and adaptive immunity has provided new opportunities to create novel and effective adjuvants based on their agonists [30]. However, the ability to induce local mucosal immune reactions when delivered parenterally was characterized in only a small number of such adjuvants [31], although many have been developed to date.

Of the more than 1000 C-type lectins, only few are type II transmembrane pattern recognition CLRs coupled to Syk and that detect pathogen-associated molecular patterns [32]. We have now investigated the impact of stimulating these CLRs (Mincle, Dectin-1, and Dectin-2) on innate as well as adaptive immunity, including both local mucosal and systemic reactions.

Syk-coupled CLRs share signaling pathways and activate a common set of several transcription factors, including NF-*κ*B, NFAT, and AP-1, which are also major regulators of immunity. Using reporter cells derived from RAW-Blue cells and which naturally express Dectin-1, Dectin-2, and Mincle, we demonstrate for the first time that stimulating these receptors with agonists like curdlan, furfurman, and TDB activates different transcriptional factors. In particular, TDB and furfurman predominantly activate AP-1 (40% and 46% of total luciferase reporter activity), while curdlan (42%) predominantly activates NFAT. Although the molecular mechanism underlying this phenomenon remains to be elucidated, the kinetics of receptor-agonist interactions, especially in *in vitro* assay conditions, are probably critical, as are the differences in downstream intracellular signals. For example, Dectin-2 and Mincle lack signal transduction motifs and require FcR*γ* to transduce activation signals [33], whereas direct Syk activation by Dectin-1 induces both canonical and noncanonical NF-*κ*B pathways [21].

The CLR agonists also activated dendritic cells, another key event in adaptive immunity, as assessed by expression of costimulatory molecules. Activation was most pronounced in response to TDB but was minimal in response to furfurman. We also found that CLR agonists induced production of Th17 cytokines from dendritic and mononuclear cells [34, 35], in accordance with the accepted model that activated dendritic cells communicate information about invading pathogens via a cytokine profile that drives Th cell differentiation. However, TDB induced maximal levels of Th1 (IL-12 p40, IL-12 p70, TNF*α*, and IFN*γ*) cytokines. On the other hand, Dectin-1 and Dectin-2 stimulation with curdlan and furfurman correspondingly predominantly induced Th17 cytokines, including IL-6, IL-23, and especially IL-17A. Moreover, we found that in response to Dectin-1 and Dectin-2 agonists, bone marrow-derived dendritic cells produced TGF-*β*1, a cytokine essential for IgA cell switching by B cells [36].

Due to these promising results, we evaluated the central and local (mucosal) immune response to ovalbumin adjuvanted with TDB, curdlan, and furfurman. While CD4^+^ and CD8^+^ T cell proliferation after *in vitro* restimulation was comparable among groups, central IgG and local IgA responses were markedly different, with TDB inducing the strongest central immune response, as indicated by total IgG, IgG1, IgG2a, and IgG2b in sera. On the other hand, curdlan and furfurman induced stronger local mucosal immune reactions than TDB, as measured by intestinal IgA. These results are consistent with the ability of furfurman and curdlan to induce, primarily, IL-17A, IL-23, and TGF-*β*1, which are central to mucosal immunity. However, curdlan and furfurman did not stimulate IgA secretion in the lung mucosa following subcutaneous immunization, in line with previous studies, in which TDB mixed with CAF01 adjuvant significantly increased lung IgA after subcutaneous/intranasal prime/boost immunization but not after subcutaneous/subcutaneous immunization [14]. These data indicate that immunization strategy also impacts mucosal immune reactions.

**Figure 8:**
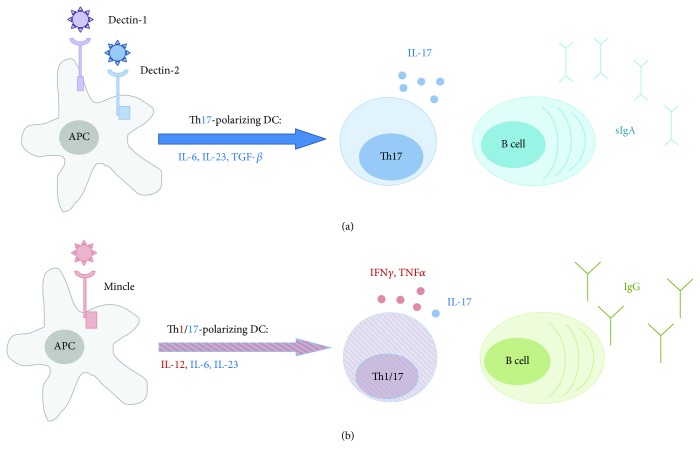
Schematic illustration of how CLR stimulation affects Th polarization and mucosal IgA production. (a) Stimulation of Dectin-1 and Dectin-2 receptors by specific PAMPs (curdlan and furfurman) results in predominant production of Th17 polarization cytokines: IL-6, IL-23, TGF-*β* by dendritic cells, and IL-17 by lymphocytes. Dendritic cells can in turn promote the development of IgA^+^ B cells in a TGF-*β*-dependent manner. Generation of mucosal IgA^+^ B cells is also favored by Th17 lymphocytes. (b) Stimulation of Mincle by TDB results in secretion of mixed Th1/Th17-polarizing cytokines by DCs (IL-6, IL-12, and IL-23). T cells are predominantly differentiated into Th1 cells secreting IFN*γ* and TNF*α*. B cells switch to producing IgG antibodies resulting in a stronger serum antibody response.

The simplified illustration summarizing differences between CLR agonists in Th polarization and mucosal IgA production is presented in Figure 8.

Finally, we showed that squalene-based formulations did not affect the IgA response to CLR agonists, suggesting that the observed effects after parenteral immunization do not depend on biodistribution or depot effects, and are mainly due to the ability of these agonists, especially curdlan and furfurman, to induce mucosal immune reactions. Along with this, we propose that inclusion of Dectin-1 and Dectin-2 agonists in vaccine composition may significantly enhance formation of mucosal IgA immune response independently from carrier nature in vaccine composition.

Taken together, the results imply that new adjuvants based on Dectin-1 and Dectin-2 agonists may significantly enhance mucosal immune reactions to newly designed or already prepared parenteral vaccines and thus may simultaneously induce both central and local immunity and confer robust and long-lived protection against mucosal pathogens. To support this assumption, further studies are required to elucidate protection efficiency of newly prepared parental vaccines containing Dectin-1 and Dectin-2 agonists as adjuvants in different experimental mucosal infections, for example, *Salmonella* and *Candida* infections. There is also important to establish potential safety pitfalls of newly identified CLR-based adjuvants. One could be expected by ability of PRR agonists to induce of systemic cytokines with unwanted side effects during parental administration.

## 5. Conclusions

In summary, we have demonstrated for the first time that despite engaging a common set of signaling pathways, stimulation of the Syk-coupled CLRs Dectin-1, Dectin-2, and Mincle using their agonists TDB, curdlan, and furfurman results in differential activation of the transcription factors NF-*κ*B, NFAT, and AP-1, maturation of dendritic cells, cytokine secretion by bone marrow-derived dendritic cells and splenic mononuclear cells, and systemic (IgG production) and local (mucosal) adaptive immunity. We have also demonstrated for the first time that stimulation of Dectin-1 and Dectin-2, but not Mincle, along with ovalbumin antigen, induces robust formulation-independent secretion of IgA in intestinal mucosa, even if delivered parenterally. Overall, this study highlights Dectin-1 and Dectin-2 agonists as adjuvants that may induce local immune reactions in intestinal mucosa and thus significantly improve the efficacy of parenteral vaccines.

## Supplementary Materials

Supplementary 1 Table S1: physico-chemical characteristics of solubilized ovalbumin, CLR agonists, and mixtures thereof. Data are mean ± standard deviation collected from three independent measurements.

Supplementary 2 Table S2: physico-chemical characteristics of vaccines formulated with squalene (*n* = 3), and loaded with ovalbumin, alone or in combination with TDB, curdlan, or furfurman. Data are mean ± standard deviation collected from three independent measurements.

Supplementary 3 Figure S1: pie charts represent differences in NF-*κ*B-, AP-1-, and NFAT-dependent luciferase reporter gene expressions in RAW-NF-*κ*B-Luc, RAW-NFAT-Luc, and RAW-AP-1-Luc macrophages, correspondingly, treated for 8 h with 20 *μ*g/mL of Mincle, Dectin-1, and Dectin-2 agonists. Each sector of the pie chart represents NF-*κ*B-, AP-1-, or NFAT-dependent luciferase reporter gene expression normalized to total luciferase expression. Stimulation of Mincle and Dectin-2 predominantly activated AP-1 (40% and 46% of total luciferase expression), whereas stimulation of Dectin-1 strongly activated NFAT (42%). In contrast, Mincle stimulation activated NFAT to a lesser extent (26%), while stimulation of Dectin-1 and Dectin-2 weakly induced NF-*κ*B-dependent expression (24% for both agonists).

Supplementary 4 Figure S2: surface plasmon resonance analysis of ovalbumin binding to TDB, curdlan, or furfurman. Letter “A” between arrows indicates time period of molecular association, and letter “D,” time period of molecular dissociation. *K*_D_ values were determined using a global fit algorithm (BIAevaluation 3.1) to be 2.35 × 10^−10^ M for TDB, 1.4 × 10^−9^ M for curdlan, and 6.3 × 10^−8^ M for furfuran. The molecular weights of TDB (987.43 Da), curdlan (1013.38 Da), and furfurman (786.65 Da) were measured on an ultrafleXtreme MALDI-TOF mass spectrometer (Bruker Daltonics, Germany). ODN1826, a CpG oligonucleotide, was used as negative control to measure nonspecific binding. Chip blank track and running buffer were not subtracted from the sensorgrams shown, which are, however, double-referenced to blank surface and blank buffer.

## References

[1] http://www.who.int/mediacentre/factsheets/fs378/en/

[2] Li W., Deng G., Li M., Liu X., Wang Y. (2012). Roles of mucosal immunity against *Mycobacterium tuberculosis* infection. *Tuberculosis Research and Treatment*.

[3] Hayashi S., Hogg J. C. (2007). Adenovirus infections and lung disease. *Current Opinion in Pharmacology*.

[4] Gralinski L. E., Baric R. S. (2015). Molecular pathology of emerging coronavirus infections. *The Journal of Pathology*.

[5] McSorley S. J. (2014). Immunity to intestinal pathogens: lessons learned from *Salmonella*. *Immunological Reviews*.

[6] Sgouras D. N., Trang T. T. H., Yamaoka Y. (2015). Pathogenesis of *Helicobacter pylori* infection. *Helicobacter*.

[7] Hebbelstrup Jensen B., Olsen K. E. P., Struve C., Krogfelt K. A., Petersen A. M. (2014). Epidemiology and clinical manifestations of enteroaggregative *Escherichia coli*. *Clinical Microbiology Review*.

[8] Darville T., Hiltke T. J. (2010). Pathogenesis of genital tract disease due to *Chlamydia trachomatis*. *The Journal of Infectious Diseases*.

[9] Doorbar J., Egawa N., Griffin H., Kranjec C., Murakami I. (2015). Human papillomavirus molecular biology and disease association. *Reviews in Medical Virology*.

[10] Schust D. J., Quayle A. J., Amedee A. M. (2012). Editorial (mucosal co-infections and HIV-1 transmission and pathogenesis). *Current HIV Research*.

[11] Freihorst J., Ogra P. L. (2001). Mucosal immunity and viral infections. *Annals of Medicine*.

[12] Lamm M. E. (1997). Interaction of antigens and antibodies at mucosal surfaces. *Annual Review of Microbiology*.

[13] Mogensen T. H. (2009). Pathogen recognition and inflammatory signaling in innate immune defenses. *Clinical Microbiology Reviews*.

[14] Christensen D., Mortensen R., Rosenkrands I., Dietrich J., Andersen P. (2017). Vaccine-induced Th17 cells are established as resident memory cells in the lung and promote local IgA responses. *Mucosal Immunology*.

[15] Mortensen R., Christensen D., Hansen L. B., Christensen J. P., Andersen P., Dietrich J. (2017). Local Th17/IgA immunity correlate with protection against intranasal infection with *Streptococcus pyogenes*. *PLoS One*.

[16] Tukhvatulin A. I., Gitlin I. I., Shcheblyakov D. V. (2013). Combined stimulation of toll-like receptor 5 and NOD1 strongly potentiates activity of NF-*κ*B, resulting in enhanced innate immune reactions and resistance to *Salmonella enterica* serovar typhimurium infection. *Infection and Immunity*.

[17] Inaba K., Inaba M., Romani N. (1992). Generation of large numbers of dendritic cells from mouse bone marrow cultures supplemented with granulocyte/macrophage colony-stimulating factor. *The Journal of Experimental Medicine*.

[18] Quah B. J. C., Warren H. S., Parish C. R. (2007). Monitoring lymphocyte proliferation *in vitro* and *in vivo* with the intracellular fluorescent dye carboxyfluorescein diacetate succinimidyl ester. *Nature Protocols*.

[19] A. I. Tukhvatulin, A. S. Dzharullaeva, N. M. Tukhvatulina (2016). Powerful Complex Immunoadjuvant Based on Synergistic Effect of Combined TLR4 and NOD2 Activation Significantly Enhances Magnitude of Humoral and Cellular Adaptive Immune Responses. *PLoS One*.

[20] Goodridge H. S., Simmons R. M., Underhill D. M. (2007). Dectin-1 stimulation by *Candida albicans* yeast or zymosan triggers NFAT activation in macrophages and dendritic cells. *The Journal of Immunology*.

[21] Gringhuis S. I., den Dunnen J., Litjens M. (2009). Dectin-1 directs T helper cell differentiation by controlling noncanonical NF-*κ*B activation through Raf-1 and Syk. *Nature Immunology*.

[22] Ishikawa E., Ishikawa T., Morita Y. S. (2009). Direct recognition of the mycobacterial glycolipid, trehalose dimycolate, by C-type lectin Mincle. *The Journal of Experimental Medicine*.

[23] de Jong E. C., Smits H. H., Kapsenberg M. L. (2005). Dendritic cell-mediated T cell polarization. *Springer Seminars in Immunopathology*.

[24] Kerscher B., Willment J. A., Brown G. D. (2013). The Dectin-2 family of C-type lectin-like receptors: an update. *International Immunology*.

[25] Kock G., Bringmann A., Held S. A. E., Daecke S., Heine A., Brossart P. (2011). Regulation of Dectin-1–mediated dendritic cell activation by peroxisome proliferator–activated receptor-gamma ligand troglitazone. *Blood*.

[26] Luckheeram R. V., Zhou R., Verma A. D., Xia B. (2012). CD4^+^T cells: differentiation and functions. *Clinical and Developmental Immunology Journal*.

[27] Rice-Ficht A. C., Arenas-Gamboa A. M., Kahl-McDonagh M. M., Ficht T. A. (2010). Polymeric particles in vaccine delivery. *Current Opinion in Microbiology*.

[28] McGhee J. R., Fujihashi K. (2012). Inside the mucosal immune system. *PLOS Biology*.

[29] Neutra M. R., Kozlowski P. A. (2006). Mucosal vaccines: the promise and the challenge. *Nature Reviews Immunology*.

[30] Steinhagen F., Kinjo T., Bode C., Klinman D. M. (2011). TLR-based immune adjuvants. *Vaccine*.

[31] Lavelle E. C., Murphy C., O’Neill L. A. J., Creagh E. M. (2010). The role of TLRs, NLRs, and RLRs in mucosal innate immunity and homeostasis. *Mucosal Immunology*.

[32] Sancho D., Reis e Sousa C. (2012). Signaling by myeloid C-type lectin receptors in immunity and homeostasis. *Annual Review of Immunology*.

[33] Kerrigan A. M., Brown G. D. (2011). Syk-coupled C-type lectins in immunity. *Trends in Immunology*.

[34] LeibundGut-Landmann S., Gross O., Robinson M. J. (2007). Syk- and CARD9-dependent coupling of innate immunity to the induction of T helper cells that produce interleukin 17. *Nature Immunology*.

[35] Robinson M. J., Osorio F., Rosas M. (2009). Dectin-2 is a Syk-coupled pattern recognition receptor crucial for Th17 responses to fungal infection. *The Journal of Experimental Medicine*.

[36] Stavnezer J., Kang J. (2009). The surprising discovery that TGF*β* specifically induces the IgA class switch. *The Journal of Immunology*.

