# Supplementary material for: Stimulation of Dectin-1 and Dectin-2 during Parenteral Immunization, but Not Mincle, Induces Secretory IgA in Intestinal Mucosa
Source: J Immunol Res. 2018 Mar 14;2018:3835720. doi: 10.1155/2018/3835720 (PMC5872666; doi:10.1155/2018/3835720)
Supplement: Supplementary 1 — Table S1: physico-chemical characteristics of solubilized ovalbumin, CLR agonists, and mixtures thereof. Data are mean ± standard deviation collected from three independent measurements. [file 3835720.f1.docx]

TABLE S1: Physico-chemical characteristics of solubilized ovalbumin, CLR agonists, and mixtures thereof. Data are mean ± standard deviation collected from three independent measurements.

|  | Size (nm) | | Z-potential (mV) | |
| --- | --- | --- | --- | --- |
|  | Mean diameter | Standard Deviation | Mean (mV) | Standard Deviation |
| ova | 11.9 | 3.4 | -26.7 | 5.5 |
| TDB | 116.2 | 23.2 | -20.9 | 4.9 |
| curdlan | 271.5 | 34.4 | -6.9 | 4.5 |
| furfurman | 106.3 | 11.0 | -12.4 | 3.7 |
| ova + TDB | 280.1 | 32.1 | -24.0 | 3.8 |
| ova + curdlan | 420.0 | 65.4 | -10.4 | 4.1 |
| ova + furfurman | 295.9 | 30.8 | -21.8 | 3.6 |
