# Supplementary material for: Stimulation of Dectin-1 and Dectin-2 during Parenteral Immunization, but Not Mincle, Induces Secretory IgA in Intestinal Mucosa
Source: J Immunol Res. 2018 Mar 14;2018:3835720. doi: 10.1155/2018/3835720 (PMC5872666; doi:10.1155/2018/3835720)
Supplement: Supplementary 2 — Table S2: physico-chemical characteristics of vaccines formulated with squalene (n = 3), and loaded with ovalbumin, alone or in combination with TDB, curdlan, or furfurman. Data are mean ± standard deviation collected from three independent measurements. [file 3835720.f2.docx]

TABLE S2: Physico-chemical characteristics of vaccines formulated with squalene (n = 3), and loaded with ovalbumin, alone or in combination with TDB, curdlan, or furfurman. Data are mean ± standard deviation collected from three independent measurements.

|  | Size (nm) | | Z-potential (mV) | |
| --- | --- | --- | --- | --- |
|  | Mean diameter | Standard Deviation | Mean | Standard Deviation |
| Sq + ova | 380.0 | 49.2 | -42.7 | 6.4 |
| Sq + ova +TDB | 363.5 | 59.8 | -35.5 | 4.8 |
| Sq + ova + Curdlan | 343.1 | 37.2 | -40.4 | 3.8 |
| Sq + ova + Furfurman | 406.4 | 69.8 | -37.2 | 4.2 |
