# Supplementary figures and images for: Stimulation of Dectin-1 and Dectin-2 during Parenteral Immunization, but Not Mincle, Induces Secretory IgA in Intestinal Mucosa
Source: J Immunol Res. 2018 Mar 14;2018:3835720. doi: 10.1155/2018/3835720 (PMC5872666; doi:10.1155/2018/3835720)

## Slide 1
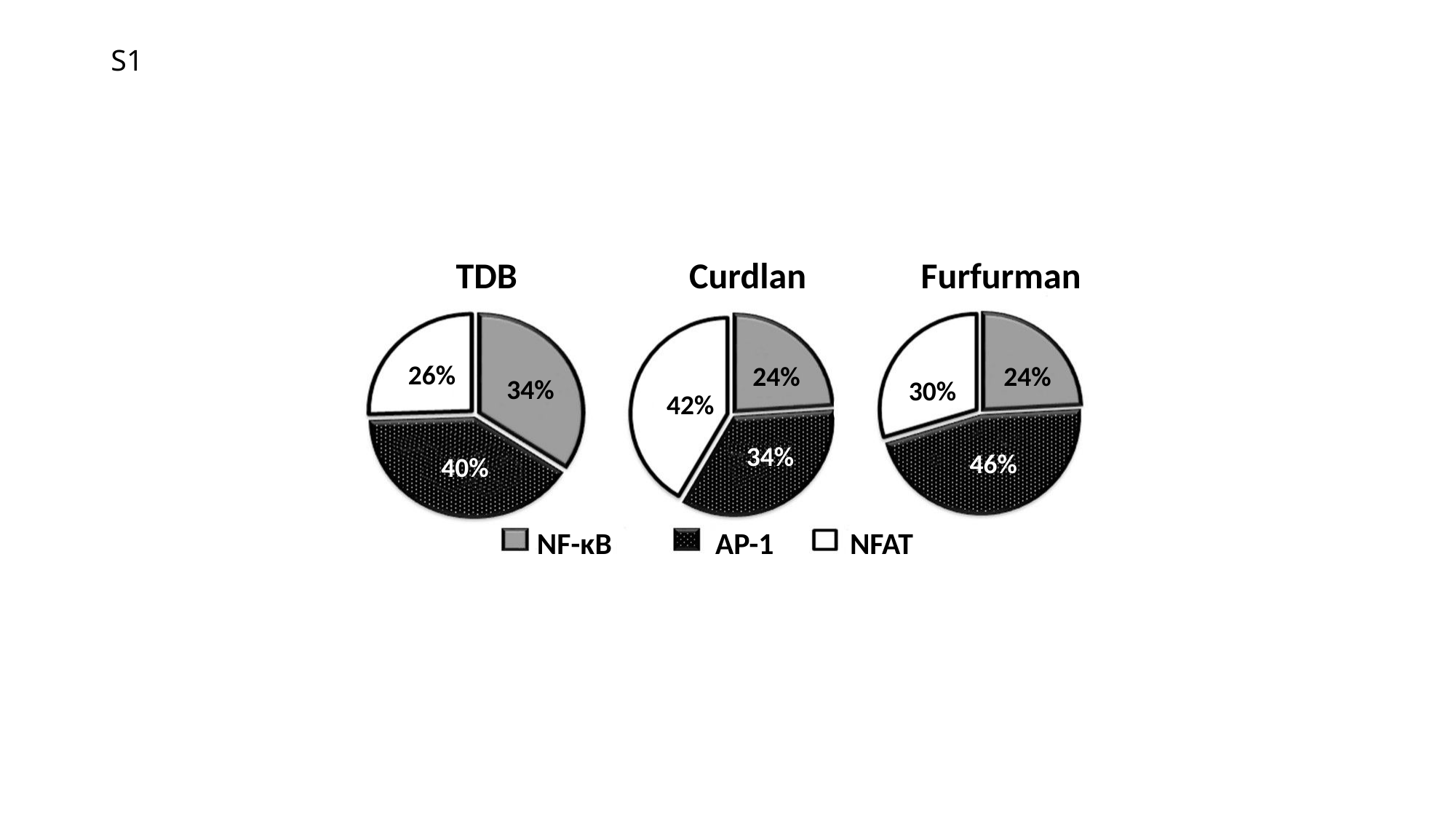

# S1
TDB Curdlan Furfurman
26%
24%
24%
34%
30%
42%
34%
46%
40%
NF-κB AP-1 NFAT

Supplement: Supplementary 3 — Figure S1: pie charts represent differences in NF-κB-, AP-1-, and NFAT-dependent luciferase reporter gene expressions in RAW-NF-κB-Luc, RAW-NFAT-Luc, and RAW-AP-1-Luc macrophages, correspondingly, treated for 8 h with 20 μg/mL of Mincle, Dectin-1, and Dectin-2 agonists. Each sector of the pie chart represents NF-κB-, AP-1-, or NFAT-dependent luciferase reporter gene expression normalized to total luciferase expression. Stimulation of Mincle and Dectin-2 predominantly activated AP-1 (40% and 46% of total luciferase expression), whereas stimulation of Dectin-1 strongly activated NFAT (42%). In contrast, Mincle stimulation activated NFAT to a lesser extent (26%), while stimulation of Dectin-1 and Dectin-2 weakly induced NF-κB-dependent expression (24% for both agonists). [file 3835720.f3.pptx]
