# Supplementary material for: Stimulation of Dectin-1 and Dectin-2 during Parenteral Immunization, but Not Mincle, Induces Secretory IgA in Intestinal Mucosa
Source: J Immunol Res. 2018 Mar 14;2018:3835720. doi: 10.1155/2018/3835720 (PMC5872666; doi:10.1155/2018/3835720)
Supplement: Supplementary 4 — Figure S2: surface plasmon resonance analysis of ovalbumin binding to TDB, curdlan, or furfurman. Letter “A” between arrows indicates time period of molecular association, and letter “D,” time period of molecular dissociation. K D values were determined using a global fit algorithm (BIAevaluation 3.1) to be 2.35 × 10^−10^ M for TDB, 1.4 × 10^−9^ M for curdlan, and 6.3 × 10^−8^ M for furfuran. The molecular weights of TDB (987.43 Da), curdlan (1013.38 Da), and furfurman (786.65 Da) were measured on an ultrafleXtreme MALDI-TOF mass spectrometer (Bruker Daltonics, Germany). ODN1826, a CpG oligonucleotide, was used as negative control to measure nonspecific binding. Chip blank track and running buffer were not subtracted from the sensorgrams shown, which are, however, double-referenced to blank surface and blank buffer. [file 3835720.f4.pptx]

## Slide 1
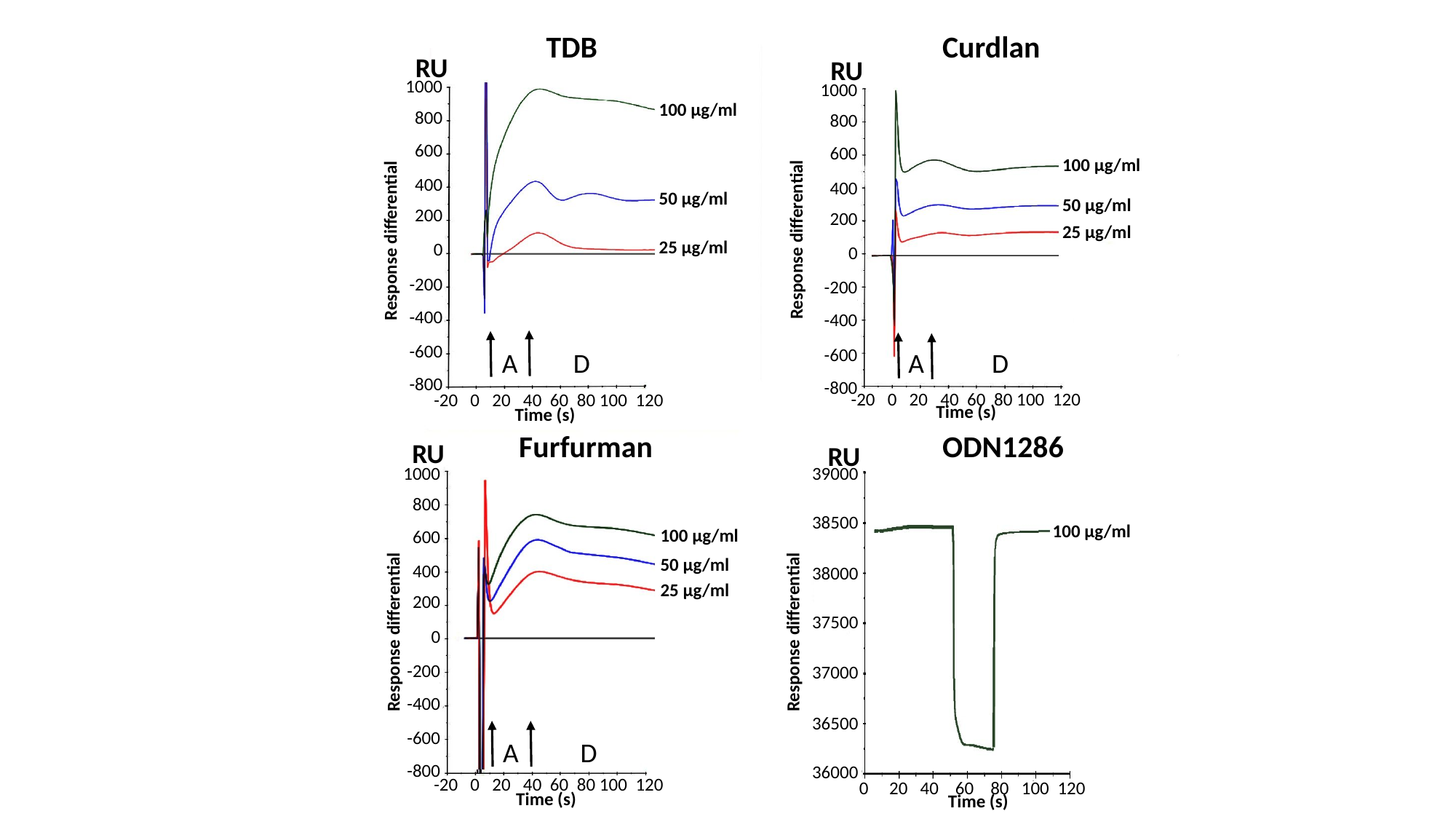

TDB Curdlan
RU
RU
Furfurman ODN1286
RU
RU
1000
800
600
400
200
0
-200
\
-400
-600
-800
1000
800
600
400
200
0
-200
\
-400
-600
-800
100 µg/ml
50 µg/ml
25 µg/ml
100 µg/ml
50 µg/ml
25 µg/ml
Response differential
Response differential
A D
A D
-20 0 20 40 60 80 100 120
-20 0 20 40 60 80 100 120
Time (s)
Time (s)
1000
800
600
400
200
0
-200
\
-400
-600
-800
39000
38500
38000
37500
37000
36500
36000
100 µg/ml
100 µg/ml
50 µg/ml
25 µg/ml
Response differential
Response differential
A D
-20 0 20 40 60 80 100 120
 0 20 40 60 80 100 120
Time (s)
Time (s)
